# Toxoplasmosis, Pancreatitis, Obesity and Drug Discovery

**Published:** 2014-09

**Authors:** Helieh S. Oz

**Affiliations:** University of Kentucky Medical Center, USA

**Keywords:** Toxoplasmosis, Oocysts, Chorioretinitis, Chemotherapy

## Abstract

Toxoplasmosis, an infectious and inflammatory syndrome, is one of the most important foodborne diseases causing hospitalization and death in U.S.A. *Toxoplasma* infects nucleated cells including pancreatic and destroys the β cells. *Toxoplasma* is a Category B classified infection by CDC and NIH, which once infected the organisms reside in tissues in cysts form for the host’s lifelong awaiting reactivation. Congenital toxoplasmosis occurs by transplacental transmission during maternal infection or reactivation of organisms and manifests with spontaneous abortion, or severe physical and mental defects. Currently, there is no safe and effective therapeutic modality against congenital toxoplasmosis or the persistent chronic infection. Here, toxoplasmosis and possible involvement of infection in induction of pancreatitis, and an experimental drug efficacy is discussed.

## Introduction

Cosmopolitan toxoplasmosis is the 3rd most important foodborne disease causing hospitalization and death in U.S.A. About 1.5 billion people worldwide are predicted to be infected with *Toxoplasma* with severe or unknown consequences. Toxoplasmosis is an infectious and inflammatory syndrome associated with the rural farming area and poverty as well as urban regions, while most cases remain undetected or misdiagnosed. An estimated 1,500,000 cases of toxoplasmosis occurs in the U.S.A alone each year, and only 15% of these cases show clinical symptoms or diagnosed [1,2]. *Toxoplasma* is a Category B classified infection by CDC and NIH, which once infected the organisms reside in muscles and brain in cyst forms for the host’s lifelong waiting for reactivation. *Toxoplasma* is an apicomplexan protozoa with sexual stage taking place in the cats’ intestinal epithelia where organisms replicate and mature to form resistant oocysts passed in the feces. Humans and animals acquire systemic form of infection in asexual stage of organism life cycle through consumption of contaminated raw meat or the mature oocysts in water and vegetables. The organisms are detected by the immunohistochemical staining or PCR methodology. Current diagnosis of infection mainly relies on serological assays to detect the presence of IgM and IgG anti-*Toxoplasma* antibodies and molecular technology. Congenital toxoplasmosis occurs by transplacental transmission of organisms during maternal infection or reactivation and manifests with spontaneous abortion, fetal death or severe defects, including encephalitis, mental illnesses, and chorioretinitis. Toxoplasmosis occurs in immunocompetent and more severe in immunosuppressed or organ transplant patients.

Organ transplant patients including pancreas and kidney recipients are at risk for toxoplasmosis as a result of immunosuppressive chemotherapy and contaminated organ or reactivation of chronic infection followed by high mortality rate if not treated. It is usually discovered in autopsy or remains undetected due to the non-specific symptoms and health care’s lack of clinical awareness [3]. Toxoplasmosis can manifest with clinical symptoms of acute or persistent abdominal pain and pancreatitis [4-6]. Chronic progressive pancreatitis may be associated with fat necrosis, inflammation and obstruction of bile duct, and fatty degeneration. Other symptoms include focal hepatic necrosis, elevated serum amylase and lipase values, and increased abdominal fat with yellowish plaques formation. Pancreas becomes enlarged and firm in palpation, white in colour appearance, and forms adhesions to the adjacent tissues. The gall bladder becomes distended with pale colour bile content. The bile duct remains tortuous and dilated and small hepatic bile ducts appear prominent. *Toxoplasma* organisms are present and may be detected in the pancreatic tissues, acinar cells and bile duct epithelial cells [7,8].

Organisms may directly attack and undermine pancreatic tissue. They may destroy the β cells and secretion of insulin and increase the risk of acute and chronic pancreatitis as well as diabetes. In a case-control clinical trial, 184 sera from diabetic and non-diabetic controls were investigated. The prevalence of anti-*Toxoplasma* IgG antibodies was respectively 61% in diabetic patients and 38% in healthy controls. Therefore, the risk factor for *Toxoplasma* infection in diabetic patients was about two folds higher than in healthy controls (RR=2.21, 95% CI; 1.6 - 3.7, P=0.001) [9]. Consequently, toxoplasmosis patients may be more at risk to develop diabetic than uninfected individuals. Indeed, insulin is shown to have a stimulatory effect on the reproduction of *Toxoplasma* organisms. While, Insulin and D-glucose have a dose-responsive mitogenic effect on the replication and development of the organisms, combined insulin and D glucose result in a synergistic stimulating effect on the intercellular *Toxoplasma* growth and replication in the cells [10]. In addition, cases of diabetes insipitus have been reported with altered neurohormonal regulation in patients with persistent or congenital toxoplasmosis [11-14].

Furthermore, toxoplasmosis may be associated with obesity or anorexia by alteration of inflammatory fat distribution as organisms alter and reside in fatty tissues [15]. However, no association was reported to link anti-*Toxoplasma* IgG antibody and obesity in 985 individuals examined from Iceland, Sweden and Estonia [16]. In contrast, excessive gestational weight gain was reported during pregnancy in infected women with *Toxoplasma* compared with uninfected pregnant women [17,18]. In addition, excess weight gain was detected in a murine pregnancy model that mimics toxoplasmosis complications in humans [19]. Similarly, infection caused increased weight gain and atrophy of myenteric neurons of the jejunum in infected animals [20]. Moreover, lipoprotein lipase is known to regulate the plasma triglyceride clearance and the distribution among organs. It is a major determinant of infection-induced hypertriglyceridemia, by modulating adipose and muscle lipoprotein lipase activity in toxoplasmosis [21]. *Toxoplasma* organisms may modulate weight gain by decreasing muscle lipoprotein lipase and altering tissue lipoprotein lipase activity during chronic toxoplasmosis to elevate triglyceride distribution in the adipose tissues.

Another factor is lipoxins effects on the host parasite control and protection against toxoplasmosis [22]. Sera levels of lipoxin A4 rise during infection and remain high in chronic infection but lack of lipoxin A4 increases mortality following infection which may be cytokine mediated tissue injury [23,24]. Additionally, *Toxoplasma* lacks the ability to synthesize sterol for growth and replication and depends on the host blood cholesterol by scavenging the low-density lipoprotein receptors. Yet, the infection elicits an exaggerated Th1 systemic inflammatory response enhancing pro-atherogenic effects [25,26].

While, inflammation is required for the host defense to protect against pathogens, non-resolving and excessive inflammatory response caused by the organisms damages the host’s cells and organs. In fact, *Toxoplasma* has been implicated in a numerous autoimmune disorders including thyroid disease, systemic sclerosis, rheumatoid arthritis, and inflammatory bowel disease, with no available cure [15].

Currently, there is no safe and effective therapeutic modality against chronic infection or congenital toxoplasmosis. *Toxoplasma* contains a rudimentary plant chloroplast epitope not present in mammals. This unique organelle has a high sensitivity to herbicide related compounds to make their use safe and attractive in infected humans and animals. A recent article by Oz and Tobin [27] using a murine fetal-maternal model of toxoplasmosis induced inflammatory syndromes to investigate safety and efficacy of Diclazuril. Diclazuril is an herbicide based agent used against coccidiosis in poultry and livestock industry. Of interest, Diclazuril treatment protected dams against excess pathological weight gain, gastroenteritis, hepatitis, pancreatitis, and splenitis. Diclazuril also, protected their nested fetuses from consequences of toxoplasmosis complications. Diclazuril therapy was shown to be safe with no detectable side effects in dams and their fetuses. This promising study warrants further investigations including future trail for the use of Diclazuril and related compounds in humans.
